# Molecular basis of a novel renal amyloidosis due to N184K gelsolin variant

**DOI:** 10.1038/srep33463

**Published:** 2016-09-16

**Authors:** Francesco Bonì, Mario Milani, Riccardo Porcari, Alberto Barbiroli, Stefano Ricagno, Matteo de Rosa

**Affiliations:** 1 CNR Istituto di Biofisica, c/o Dipartimento di Bioscienze, Università degli Studi di Milano, 20133 Milano, Italy; 2 Dipartimento di Bioscienze, Università degli Studi di Milano, 20133 Milano, Italy; 3 Wolfson Drug Discovery Unit, Centre for Amyloidosis and Acute Phase Proteins, University College London, London NW3 2PF, UK; 4 Dipartimento di Scienze per gli Alimenti, la Nutrizione e l’Ambiente, Università degli Studi di Milano, 20133 Milano, Italy

## Abstract

Mutations in gelsolin are responsible for a systemic amyloidosis first described in 1969. Until recently, the disease was associated with two substitutions of the same residue, leading to the loss of the calcium binding site. Novel interest arose in 2014 when the N184K variant of the protein was identified as the etiological agent of a novel kidney-localized amyloidosis. Here we provide a first rationale for N184K pathogenicity. We show that the mutation induces a destabilization of gelsolin second domain, without compromising its calcium binding capacity. X-ray data combined with molecular dynamics simulations demonstrates that the primary source of the destabilization is a loss of connectivity in proximity of the metal. Such rearrangement of the H-bond network does not have a major impact on the overall fold of the domain, nevertheless, it increases the flexibility of a stretch of the protein, which is consequently processed by furin protease. Overall our data suggest that the N184K variant is subjected to the same aberrant proteolytic events responsible for the formation of amyloidogenic fragments in the previously characterized mutants. At the same time our data suggest that a broader number of mutations, unrelated to the metal binding site, can lead to a pathogenic phenotype.

Gelsolin is a 80 kDa protein displaying six homologous, gelsolin-like, domains (named G1 to G6) existing as two alternative splicing variants: a 24-residue shorter intracellular form and a variant secreted in the bloodstream^1^,^2^. Both isoforms bind actin fibrils, severing the filaments and preventing repolymerization. Gelsolin is the prototype of the gelsolin superfamily, whose members are all multidomain proteins, highly regulated by calcium. Gelsolin, in particular, hosts eight calcium binding sites: one for each domain and two incomplete sites, which require actin contribution. In the absence of the ion, gelsolin assumes a compact conformation, in which domains G1–G5 are wrapped around G6^2^, masking the actin binding sites (inactive conformation). Upon increase of the ion concentration, gelsolin undergoes major conformational changes, with large interdomain rearrangements. Such molecular gymnastic is believed to happen in a sequential and cooperative fashion, ultimately leading to the open and active configuration. Active gelsolin eluded structural investigations, most probably due to its highly dynamic nature. In fact, recent studies on adseverin showed that the active conformation of gelsolin-like proteins is better described by an ensemble of structures, characterized by a larger gyration radius with respect to the inactive form^3^. Upon binding to actin, gelsolin adopts a novel conformation^2^,^4^, although the structures of the individual domains closely match those observed in the inactive form: new interdomain contacts are formed while others are lost^2^,^4^.

Due to its polyvalent function, gelsolin is somehow involved in a plethora of pathological processes, in particular various cancer types^5^. Moreover, mutations in the gelsolin gene are directly responsible for gelsolin amyloidosis (GA) with 100% penetrance. GA was first described in 1969^6^ and for long referred as familial amyloidosis Finnish-type (FAF), characterized by deposition of amyloids in different organs and tissues. The parts of the body mostly affected are the eyes, the cranial and peripheral nerves, the skin and, at later stages, the kidneys. Such heterogeneous spectrum of symptoms makes GA very difficult to diagnose.

Until recently, only two mutations were described leading to GA (D187N and D187Y). The mechanism of amyloid formation by these variants is relatively well understood, although it awaits structural confirmation. D187 is part of a cluster of residues binding the calcium ion in the G2 domain^7^. Substitutions in such position with either N or Y impair calcium binding, causing destabilization of the G2 domain^8^,^9^. Whether this destabilization is due to a conformational change of the native state or to an increased flexibility of the overall fold is not known, as the structure of the mutated proteins have not been reported^10^. In either case, in the *trans*-Golgi network (TGN), exported gelsolin encounters calcium at millimolar concentration, sufficient for the activation and opening of the protein structure. In the presence of a destabilizing mutation, protein activation leads to the exposure of the region comprising residues 168 to 173, and the subsequent proteolysis at R172 by the endogenous protease furin^11^. The C-terminal fragment, known as C68, is exported in the extracellular environment, where is subjected to further aberrant proteolytic events, eventually leading to the formation of 5 and 8 kDa peptides, which are the major constituent of aggregates in GA^12^. Such peptides harbours the mutation in position 187 and are more prone to amyloid aggregation than homologous wt fragments^13^: that is, D187N/Y substitutions play a dual role in GA pathological mechanism, both promoting aberrant proteolysis and increasing gelsolin inherent amyloidogenicity.

Recently, other two gelsolin mutants (G167R and N184K) have been identified as associated with pathologic amyloid deposition^14^,^15^. To date the deposition of such mutants seems to cause solely kidney symptoms, however, only a partial clinical picture is available and degree of penetrance of these mutations unknown. These mutations fall in the G2 domain of gelsolin and are in close proximity to the calcium binding site.

Here we report a structural and functional characterization of the isolated G2 domain of the N184K variant. We show that the mutation induces a destabilization of the protein fold without compromising the calcium binding site. Such destabilization confers high susceptibility to furin proteolysis, suggesting that the N184K variant is subjected to the same aberrant proteolytic cascade described for the mutants in position 187.

## Results

Although the isolated G2 domain is commonly accepted as a valuable system to study the effects of the pathological mutations on the structure and function of the protein, the definition of its exact boundaries has been subjected to extensive discussion. Two groups, respectively coordinated by A.R. Fersht and J.W. Kelly, mainly contributed to the dissection of the molecular bases of GA. The former group worked on a shorter construct, whose design was based on limited proteolysis studies^8^, hereafter named G2s. The latter used a longer construct suggested by the crystal structure of the Ca-free full-length protein (G2l)^10^ (see Supplementary Fig. S1). In our work, we used the G2l construct for all the experiments in solution, nevertheless crystals grew only for the G2s, consequently, when necessary, experiments were repeated on the shorter construct to validate the structural models.

### N184K mutation destabilizes gelsolin G2 fold without compromising calcium binding

Previously characterized D187N/Y mutations strongly destabilize gelsolin second domain. Such destabilization is primarily due to the calcium binding impairment, which has an important role in gelsolin activation and structural stability of the individual domains.

A preliminary evaluation of N184K G2l thermal stability was assessed by denaturation experiments in the presence of SYPRO orange fluorescent probe (see Supplementary Fig. S2) to find suitable conditions for its biochemical and structural characterization. These tests suggested that a higher ionic strength was required and that the optimal conditions for the G2l mutants were 20 mM HEPES, pH 7.0, 300 mM NaCl.

**Figure 1:**
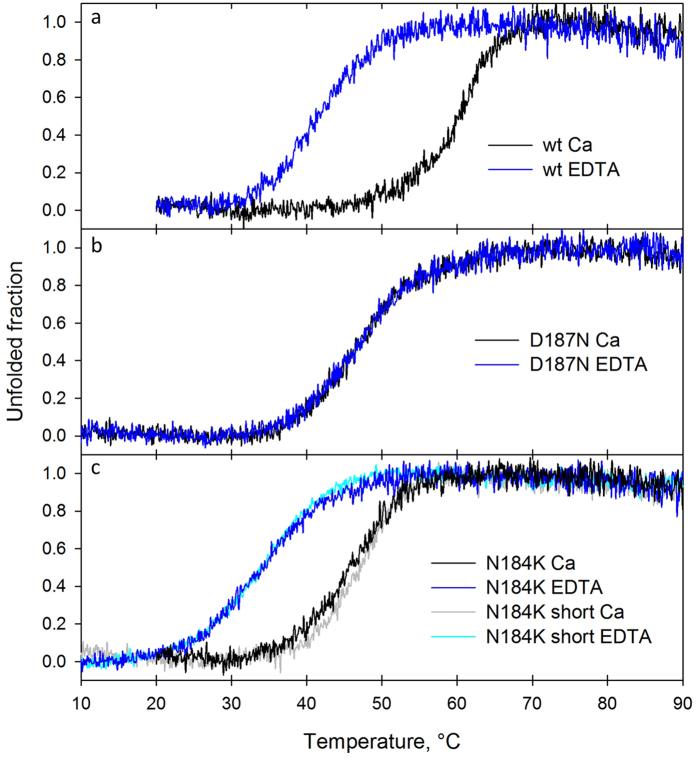
N184K mutation destabilizes gelsolin G2 fold. Thermal denaturation studies of wt, (**a**) D187N (**b**) G2l domain and both G2l/G2s construct of N184K variant (**c**). Loss of secondary structure was monitored by CD at 218 nm in the presence of either 1 mM calcium chloride (black/grey trace) or 1 mM EDTA (blue/cyan trace).

Protein unfolding was monitored by CD in the far UV region in the presence of a molar excess of either CaCl_2_ or EDTA. Our results on wt and D187N G2l well reproduce those reported by Huff *et al*.^16^. Indeed, the wt (Tm = 60.6 °C) is strongly stabilized by the presence of calcium (∆Tm = +19 °C), whereas the mutant shows Tm values very similar to the wt apoprotein (Table 1 and Fig. 1).

Surprisingly, N184K G2l variant exhibits a shift of the Tm value upon addition of a molar excess of EDTA, indicating that N184K is still able to bind Ca^2+^ and that the ion provides a significant stabilization (∆Tm = +12 °C) of the domain. Nevertheless, the mutation destabilizes the G2 fold: The Tm of N184K is 45.6 °C, 15 °C lower than the Tm measured for the wt domain.

CD spectra and temperature ramps were recorded for both G2l and G2s N184K constructs. G2l and G2s CD spectra are almost identical and Tm’s calculated in the presence of either calcium or EDTA (47.0 and 33.8 °C, respectively) are within the experimental error, suggesting that the N-terminal extension does not influence the stability of the domain nor induces detectable changes at the secondary structure level.

### N184K mutation increases gelsolin G2 susceptibility to furin proteolysis but it does not confer intrinsic amyloidogenicity

**Figure 2:**
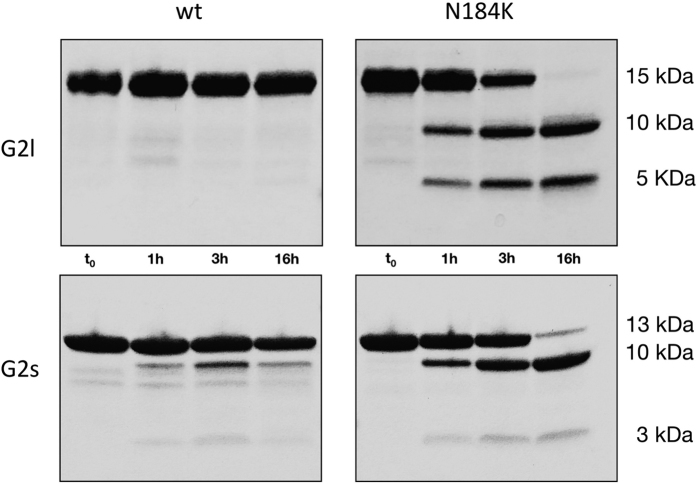
G2 N184K variant is promptly cleaved by furin. Susceptibility to proteolysis was tested *in vitro* on both the long constructs (G2l wt and N184K, top panels) and short constructs (G2s wt and N184K, bottom panels). Production of fragments of 10 and either 5 or 3 kDa are observed mainly for the N184K variant, in accordance with cleavage of the G2 domain at the furin recognition site (168-RRVVR↓A-173).

D187N/Y mutations increase isolated G2 domain sensitivity to proteolysis by furin^16^, which is believed to be the first event leading to gelsolin amyloidosis. Here, we wanted to test if the mutation in position 184 was conferring similar susceptibility. Such tests were performed at pH 6.5 and in the presence of 1 mM CaCl_2_ to mimic conditions found in the TGN and because furin is a calcium dependent protease. The reaction was followed for roughly 16 hours and samples periodically collected to define a time course of the process. N184K G2l mutant displays high susceptibility to furin aberrant proteolysis: bands corresponding to 5 and 10 kDa fragments appear after only 1 h incubation and the G2l domain is totally processesed after 16 hours. Contrary, wt G2l domain is extremely resistant to proteolysis under the tested experimental conditions (Fig. 2, top panels).

The experiment was also repeated on the wt and N184K G2s’ (Fig. 2, bottom panels). These constructs, which lack a short edge strand interacting with the β1 strand, show a pattern of proteolysis very similar to that observed for the longer forms. During the proteolysis of wt domain some faint bands, possibly product of furin activity, can be observed but their intensity is not comparable with the ones visible in the experiments with the mutant. These results suggest that the N-terminal stretch does not represent a crucial protective element from aberrant proteolysis nor, in keeping with thermal stability data, has a major impact on the fold of the domain.

In order to assess if the observed proteolytic event is due to the increased mobility of the mutated domain, we used wt and N184K G2l variants in limited proteolysis experiments with an unspecific protease. We chose trypsin for this analysis because G2 domain is particularly rich in positively charged residues, (Arg + Lys make up for more than 10% of the sequence) and a new potential cleavage site is created by the mutation itself. Nevertheless, the two variants show both an identical proteolytic pattern and a comparable kinetics of the intact domain (up to 20’), with a rapid appearance of two bands relatively stable over time (see Supplementary Fig. S3). After these early proteolytic events, further degradation appears to progress faster for the N184K fragments. This experiment further strengthens the results shown in Fig. 2, suggesting that furin cleavage is a very specific event due to an inherent susceptibility conferred by the mutation.

**Figure 3:**
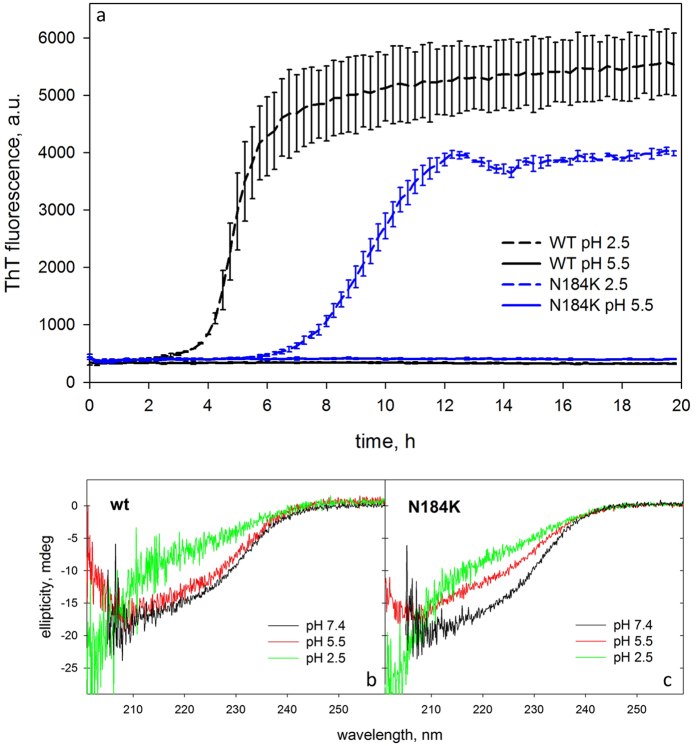
N184K mutation does not increase G2 inherent propensity to form fibrils. (**a)** Aggregation kinetics monitored by the increase of ThT fluorescence of wt (black traces) and N184K (blue traces) G2l variants at both pH 2.5 (dashed lines)and 5.5 (solid lines). (**b**,**c**) CD spectra of wt (left panel) and N184K mutant (right panel) G2l in the fibrillogenesis conditions compared to the spectrum at physiological pH.

Fibrillogenesis of wt and N184K G2l variant was carried out at both pH 5.5 and 2.5, monitoring thioflavin T fluorescence. In the former condition, no aggregation occurs for either the wt or the mutant domain. On the contrary, at pH 2.5, wt G2l shows a higher propensity for amyloidogenesis than the variant (Fig. 3a). Secondary structure content was monitored during the experimental time frame by CD spectroscopy (Fig. 3b,c). At lower pH the two variants show a spectrum consistent with a completely unfolded protein. Conversely at pH 5.5, the spectrum of wt is comparable to those registered at physiological conditions, whereas, the N184K mutant is partially denatured. We can therefore infer that the mutation does not increase the intrinsic propensity of gelsolin to aggregate under both denaturating and milder conditions.

### Structural bases of G2 N184K instability and susceptibility to aberrant proteolysis

To decipher the structural basis of N184K sensitivity to furin proteolysis, its structure was determined by X-ray crystallography. Although extensive crystal screenings were performed on N184K G2l construct, we failed to obtain crystals of suitable quality. As literature data and our limited proteolysis experiments suggest, the flexible N-terminal loop could prevent the growth of well-ordered crystals, thus we resorted on the use of the shorter G2s construct, lacking this N-terminal stretch.

Crystals of N184K G2s displaying two different morphologies readily appeared in different conditions. Best diffracting crystals were used to determine N184K structure at 1.05 and 1.7 Å resolution, which will be hereafter referred as the orthorhombic and trigonal forms, respectively (Table 2). Electron density was of excellent quality for both structures and allowed the tracing of all residues belonging to the core of the G2 domain. Only few amino acids were not modeled: residues 260–266 at the N-*terminus* of the orthorhombic crystal form, and residues 256–266, 158–159 and the side chain of 169 in the trigonal crystal. In both crystal forms the prototytipical gelsolin-like fold, made of a 5-strand β-sheet sandwiched between two α-helices is maintained.

**Figure 4:**
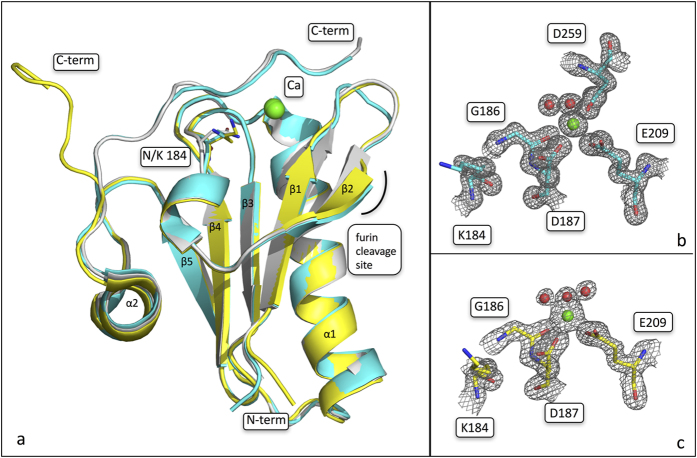
Impact of N184K mutation on the overall fold of gelsolin G2 domain. (**a**) Superimposition of the crystal structures of N184K (orthorhombic and trigonal crystal forms, coloured in cyan and yellow, respectively) and wt protein (pdb id 1KCQ, in light gray). Residue 184 is shown as sticks and labeled; N- and C-*termini*, the calcium ion and the aberrant protelytic site are also marked. (**b**,**c**) Details of the calcium binding site in the two N184K structures; the 2Fo-Fc electron density map of the residues and the water molecules coordinating the ion are shown as a grey grid countured at 1.5 σ.

In agreement with denaturation studies, electron density for Ca^2+^ was clearly visible in its binding site and the ion was refined to 100% occupancy in both structures (Figs 4 and S4). In addition, other ions were found coordinated between symmetric molecules, likely contributing to the formation of the tight crystal packing, which lead to the high resolution diffraction achieved but irrelevant for the structure of G2.

Visual inspection of a superimposition of the two structures with the wt G2 domain (pdb id 1KCQ), highlighted some remarkable differences in the trigonal crystal, whereas the mutation seems not to have a major impact on the domain in the orthorhombic form (Fig. 4). In the wt structure, the C-terminal tail is wrapped around the globular domain and is involved in extensive interactions with loops β2-β3 and β1-β2. More importantly, it provides a sort of lid for the calcium binding site, contributing to the hexacoordination of the ion with the side-chain of D259. In contrast, in the trigonal crystal the C-*terminus* dissociates from the Ca^2+^ coordination, assuming a stretched configuration (Fig. 4). Such conformational change leaves the calcium ion solvent exposed, with a water molecule taking the place of the carboxylate of D259. Nevertheless, the calcium ion seems to be still firmly bound to the protein, as its B factor is comparable to those of the surrounding atoms.

Beside the C-terminal tail, the two N184K crystal forms match each other very closely, as highlighted by a Cα r.m.s.d. value of 0.25 Å (residues 160–250). A similar Cα r.m.s.d. value (0.56 Å for 102 atoms) can be calculated between the mutant in the close conformation and the wt structure.

The open conformation of the C-*terminus*, observed in the trigonal crystal, is likely due to crystal packing: the terminal tail interacts with residues of the N-terminus and of the β3-β4 loop belonging to a symmetric molecule (Fig. S5). Therefore, we focused on the orthorhombic form. In this structure the mutation seems to have only a minor impact on the overall structure of the G2 domain. Interestingly, the experimental electron density of the β1-β2 turn is of lower quality with respect to the rest of the protein (Fig. S4), displaying B factors significantly above the average. Such loop (residues 167–170) is part of the aberrant proteolytic site (168-RRVVR↓A-173) and its enhanced motility could explain the higher susceptibility to furin proteolysis displayed by the mutant. Another area of the protein characterized by relatively high B factors and poor electron density is the hinge region following the α2 helix.

**Figure 5:**
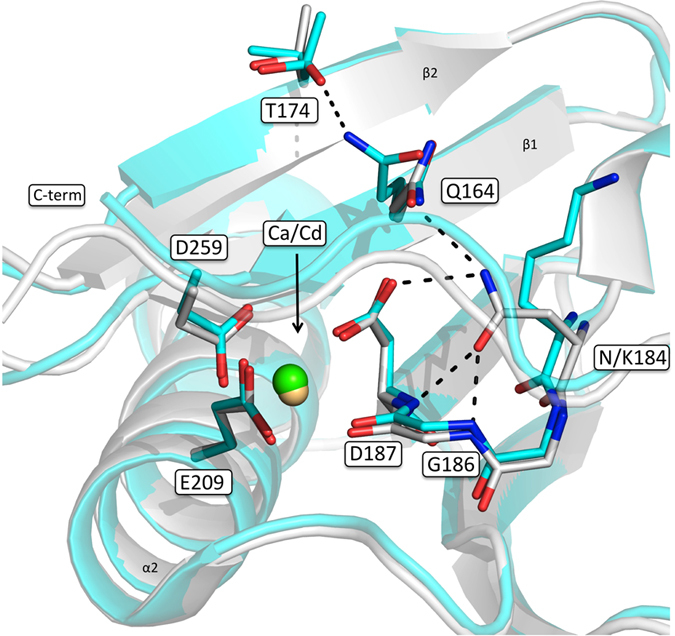
N184K mutation perturbs the H-bond network around Ca^2+^ binding site. N184K structure (orthorhombic crystal; cyan) overlaid onto the wt protein (white). N/K184, the residues forming the calcium binding site, N164 and T174 are shown as sticks and labeled. Polar interactions between N184 and N187, G186 and Q164, which are lost upon mutation, are highlighted by dashed lines as well as the new interaction between Q164 and T174.

N184 does not directly coordinate the calcium ion, nevertheless it is hydrogen bonded to D187 (Fig. 5), the residue mutated in FAF, whose substitution to either N or Y leads to the loss of calcium binding capability. In the variant object of this study, not only the calcium is firmly bound but also the geometry of the coordination site matches perfectly the one observed in the wt structure. In the mutant, N184 is substituted with a bulkier lysine, which does not fit in the pocket occupied by the asparagine. As a result, the long flexible side-chain of lysine protrudes toward the solvent, without causing a significant rearrangement of the neighboring residues. Nevertheless, the mutation induces a weakening of a H-bond network around the Ca^2+^ coordination site involving mainly four residues: Q164, N184, G186 and the Ca^2+^ bound D187. In details, N184, in the wt protein, is H-bonded to the N atoms of both G186 and D187 and to one of the Oδ atoms of the latter (Fig. 5). All the above mentioned interactions are lost in the mutant. In addition, two longer-range polar contacts can be observed between Q164 and both D187 and N184 in the wt protein. In the mutant, Q164 is present in two alternative conformations correlated with the two conformations of T174 (refined to the same occupancy; Fig. 5): when Q164 looses the interaction with D187 it is H bonded to T174.

**Figure 6:**
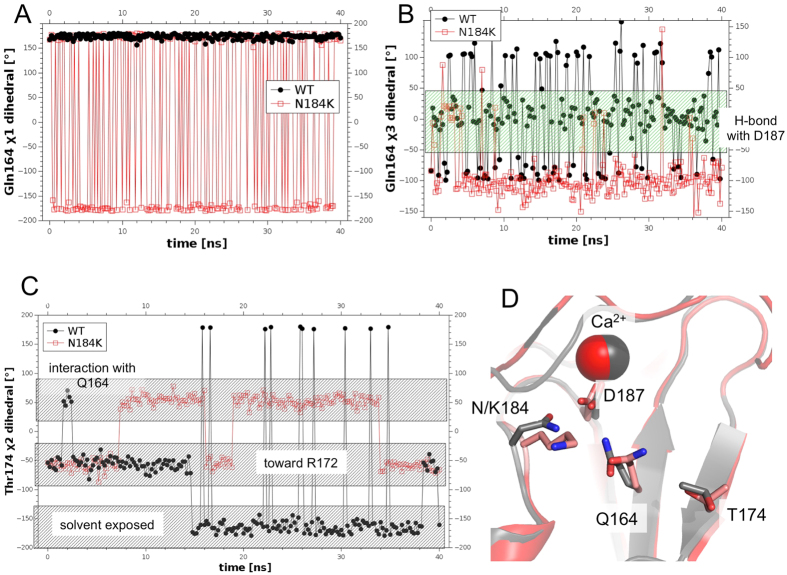
Molecular dynamics simulation of WT and N184K mutant. (**A**) χ1 dihedral of Q164 in wt (black dots) and N184K variant (open red squares) is stable in wt and oscillates between two states in the mutant. (**B)** χ3 dihedral of Q164 shows higher propensity to form H-bond with D187 (values between −50 and 50) in the wt (black dots) respect to the mutant (open red squares). (**C**) T174 χ2 dihedral in N184K (open red squares) and wt (black dots) shows a higher propensity for the mutant to establish H-bond with Q164 (values between 10 and 90). (**D)** Superimposition of the wt (gray) and N184K variant (red/pink) after 30 ns of MD simulation: the reorientation of Q164 causes a loss of H-bond with D187 and a gain of H-bond with T174.

In the orthorhombic structure an interesting gain of motility in a region close to the furin cleavage site was observed. In order to better characterize it, we decided to perform two molecular dynamics simulations on the wt and the N184K mutant. To minimize all other possible effects except those induced by the mutation we chose to start both simulations from the same coordinates of the orthorhombic crystal structure, after the addition of the missing terminal amino acids (modeled in random conformation) with either asparagine or lysine in position 184. Overall the mutated protein displays an enhanced motility during the simulation as shown by the Cα root mean square fluctuation (RMSF, *i.e*. standard deviation) of atomic positions along the trajectory (see Supplementary Fig. S4). Consistently with the crystallographic analysis, the side chain of K184 points toward the solvent during all the 40 ns simulation, allowing a greater conformational freedom for Q164 with respect to the wt protein (Fig. 6A). Accordingly, in the mutant protein Q164 is involved in H-bond with D187 only in 10% of the simulation time (the mean distance between the two residues being 4.36 Å) whereas in the wt the two amino acids remain closer (mean distance = 3.67 Å) establishing H-bond for about 54% of the simulation time (Fig. 6B). The different conformation of Q164 directly affects the dihedral of T174 side chain (Fig. 6C). In conclusion, the overall effect of the N184K mutation is transmitted by Q164 toward the furin cleavage site, causing a greater conformational freedom of β1-β2 loop respect to the wt (Fig. S4). Such distinct dynamical features might be the key factors in promoting the efficient furin binding and processing.

## Discussion

Gelsolin amyloidosis is a relatively rare genetic disease caused by accumulation of aggregated proteolytic fragments of the protein gelsolin. The Finnish form of this malady (FAF) has been known for long time and it is due to mutation of aspartate 187 in either asparagine or tyrosine in the G2 domain of the protein. These variants are unable to bind calcium, which plays a stabilizing role, resulting in a misfolded protein prone to aberrant proteolysis.

Recent development of diagnostic tools for the identification and typing of amyloidosis^17^ promptly led to the identification of two novel variants of gelsolin carrying amyloidogenic mutations^14^,^15^: N184K and G167R, suggesting that GA might be more common than it is believed to date. Interestingly, both mutations fall in the G2 domain and are sited in close proximity to the calcium binding site. Amyloid fibers arising from these variants, seem to accumulate solely in the kidney, whereas mutants in position 187 lead to a more systemic amyloidosis characterized by the symptomatic triad: corneal lattice dystrophy, peripheral polyneuropathy and cutis laxa. The determinants of kidney localized deposition are not known nor whether is the full-length protein or fragments forming the fibrillar tangles, as only a partial clinical description of these variants is available so far. For these reasons we focused on the molecular characterization of N184K G2 domain, to gain insights into the pathogenicity of this variant.

Our results show that N184K severely affect gelsolin stability, as it is the case of FAF and many other monogenic diseases^16^,^18^. At the same time the mechanism behind such destabilization is subtler. In fact, the N184K variant is still able to bind calcium and the ion affects the domain stability in a similar way as observed for the wt. This result is of a pivotal importance, suggesting that a broader number of potential mutations may lead to a pathogenic phenotype. Interestingly, the primary source of the N184K destabilization is a rearrangement of the H-bond network around the calcium binding site. K184 side chain is found extruded toward the solvent in the crystal structures and throughout the MD simulation, consistent with a permanent loss of the interactions that in the wt domain involve the lost asparagine. At the same time, the mutation, although without a major impact on the fold, affects the overall motility of the domain and in particular that of the β1-β2 loop (some 20 Å afar). Such increased dynamicity is observed both in the crystallographic structure, in terms of higher average B factors, and in the simulations monitoring the r.m.s.f. values (Fig. S4). The effects of the mutation are transmitted toward the β1-β2 loop by Q164 side chain (in β1), that loosing the permanent H-bonds with D187 and N184, can oscillate between two conformations (Figs 5 and 6A). In one of the two conformations Q164 interacts with T174 (in β2) downward to the furin cleavage site (168-RRVVR↓A-173). Interestingly, even if in both wt and N184K mutant the region around β1-β2 loop displays higher B factor values respect to the average, such effect is larger in the mutant: 38.9% growth vs. 28.2%.

Similar results were obtained by NMR and MD studies^7^,^9^ on the D187N mutant where, in particular in the latter, Kazmirski and co workers not only observed a very mobile β1-β2 loop but even the loss of structure of the flanking strands by the end of the MD simulation^9^. Such destabilization was mainly caused by the loss of the salt bridge between D187 and K166, with the latter residue playing a similar role in briging the mutation site to the β1-β2 loop as observed for Q164 in the N184K variant.

In addition, Kazmirski *et al*., noticed a significant increase in the C-terminal tail dynamics, which represents a protective element from furin proteolysis due to its steric hindrance. In one of our structure we found such tail in an *open* conformation, (not observed during our MD simulation), suggesting that even in the N184K mutant this stretch of the protein is more flexible and prone to unfolding. Overall, the comparison of D187N and N184K indicates that both mutations have an impact on the dynamical properties of the protein although to different extent.

Indeed, the aforementioned β1-β2 loop corresponds to the stretch of protein recognized by furin, comprising residues 168–173, and we showed that N184K mutant is promptly processed by the protease and that this event is due to specific recognition. In fact, experiments using trypsin did not show a generic susceptibility to proteolysis of the mutant, if the sensitivity of the intact domain is compared, as the reduced stability measured by thermal unfolding could have suggested. This result is in keeping with the structural analysis where we observe subtle differences only in that specific region of the G2 domain. Although furin assays suggest that N184K variant is subjected to the same proteolytic cascade of the FAF mutants, we tested if the mutation could tune the aggregation propensity of the intact G2 domain. Indeed, the gelsolin mutations in position 187 not only are responsible for the aberrant proteolytisis but also the fragments formed, which contain the mutation, are more prone to aggregate compared to an equivalent wt peptide^13^. Moreover, aggregation efficiency is pH dependent and increases at lower values^12^. Our fibrillogenesis experiments in both nearly physiological and denaturant conditions showed that, while at pH 5.5 neither the wt protein nor the mutant aggregates, at low pH, when the G2 domain is totally unfolded, aggregation occurs. Unfolding leads to the exposure of gelsolin amyloidogenic core, which has been identified as the peptide spanning residues 179–194^19^. Interestingly, N184K mutant shows both a later onset and a longer lag phase of fibril formation. Although confirmation would come from repetition of the experiment on the protelytic fragments, which is beyond the focus of this work, these results show that the N184K mutation does not increase the intrinsic amyloid propensity of the G2 domain. Moreover, our results suggest that the amyloidogenic peptides carrying the N184K mutation would be less prone to aggregation respect to the wt, which well correlates with the milder phenotype of the disease, not systemic but localized in kidneys.

An additional problem we had to face during the characterization of N184K G2 domain was the role of the N-terminal stretch comprising the short edge strand (β_0_) and the loop connecting the same to β1 or, in other words, which is the best definition of gelsolin domain 2. The issue has been already extensively discussed^7^,^16^ but what we believe to be the key experiment, i.e. furin proteolysis assay, was never performed in parallel on the two constructs. Our results clearly show that the β_0_ strand does not protect G2 domain from aberrant proteolysis. Therefore, the G2s construct is an appropriate and convenient model for the biochemical characterization of gelsolin G2 domain, with the added value to be suitable for crystallographyc studies.

In conclusion, our results show that the N184K mutation does not have a major impact on the fold of the G2 domain, while it affects the mobility of the β1 strand and of the loop downstream. Such clear increase of dynamicity promotes the docking of furin protease, which cleaves the protein with comparable efficiency respect to the mutants in position 187^16^. Although we do not have data in regards, we can speculate that the cleaved fragment would be further proteolysed by enzymes from the extracellular matrix with the formation of the 5 and 8 kDa peptides carrying the N184K mutation. These peptides seem to be less prone to aggregation than those originated from the 187 mutations, leading to a milder phenotype that causes a localized deposition and not a systemic disease. In this context the N184K mutation could be nearly considered a risk factor rather than a pathogenic mutation, but more clinical and molecular data are required to have a complete picture of the disease.

## Methods

### Protein production

#### Cloning, mutagenesis and expression

Synthetic gene coding for the second domain of human gelsolin (G2l, residues 133-266) was purchased from Eurofins Genomics and subcloned into a pET-28 plasmid at the Nde I/Xho I sites (Novagen) carrying a 6xHis-tag at the N-terminus.

G2l mutants (D187N and N184K) were produced by site-directed mutagenesis using G2l wt as template and the phusion mutagenesis kit (Thermofisher). The shorter construct of N184K (G2s, res 151-266) used for crystallographic studies was also obtained by site-directed mutagenesis according to the two-step protocol described in ref. 20.

Recombinant plasmids (G2l_wt/pET28, G2l_D187N/pET28, G2l_N184K/pET28 and G2s_N184K/pET28) were transformed in the *E. coli* strain BL21 pLys (DE3) (InvitrogenTM). Cell cultures were grown in LB Broth at 37 °C. Expression of G2l and G2s was induced by addition of 1 mM IPTG.

#### Purification

Typically, 10–20 mg of cells were resuspended in lysis buffer (20 mM sodium phosphate, pH 7.4, 0.5 M NaCl, protease inhibitors (cOmplete, EDTA-free, Roche), 10 μg/ml of Deoxyribonuclease I) and lysed by sonication. The clarified crude extract was incubated with 2 ml of Ni^+2^ resin (Ni SepharoseTM 6 Fast Flow, GE Healthcare) for 2 hours at 4 °C, and after extensive washing with the lysis buffer, G2l protein was eluted with 20 mM NaPO_4_, 0.5 M NaCl, 0.3 M Imidazole.

After cleavage of the 6xHis-tag (5 U of thrombin/mg of G2 for 1 h at room temperature) the sample was passed through a HiLoad 16/600 SuperdexTM75 column equilibrated with 20 mM HEPES, 300 mM NaCl, pH 7.4.

The N184K G2s variant was further purified on a MonoQ 5/50 GL column equilibrated with 10 mM TrisHCl, 20 mM NaCl, pH 8.5 and eluted with a linear NaCl gradient (from 20 mM to 1 M).

### Thermal stability

#### Thermofluor assay

The experiments were carried out using a MJMini™ Personal Thermal Cycler (BIO-RAD), heating the samples from 10 to 99 °C. Fluorescent Sypro Orange (Sigma) was used to monitor G2l thermal denaturation. 2.5 μl of G2l (final protein concentration 5 mg/ml) were mixed with 3.5 μl of a 1:500 dilution of Sypro Orange commercial solution and 19 μl of screen solution. 15 μl of each final solution were used to perform the ThermoFluor assay. Thermal stability was evaluated as function of the pH and NaCl concentration: 20 mM HEPES pH 6.5/7.0/7.5, NaCl 0/100/300 mM, with either 1 mM CaCl_2_ or 1 mM EDTA. The fluorescence intensity was measured (ΔT = 0.2 °C) within the excitation/emission wavelength ranges of 470–505/540–700 nm.

#### Circular dichroism

CD measurements were performed with a J-810 spectropolarimeter (JASCO Corp., Tokyo, Japan) equipped with a Peltier system for temperature control. All measurements were performed in 20 mM HEPES, 100 mM NaCl, pH 7.4 at 0.2 mg/ml protein concentration. All temperature ramps were recorded from 10 to 95 °C (temperature slope 50 °C/hour) in a 0.1 cm path length cuvette and monitored at 218 nm wavelength. Tm was calculated as the minimum of the first-derivative of the traces. Spectra before and after unfolding ramp were recorded (260–190 nm).

### Furin assays

Furin cleavage assays were performed using 1 U of commercial furin enzyme (BioLabs) and 1 mg/ml of G2l/G2s variants in 20 mM MES, pH 6.5, 100 mM NaCl, 1 mM CaCl_2_. The final volume of each reaction mix was 50 μl and the reaction was performed at 37 °C. To monitor the reaction, 12 μl aliquots of the reaction mix were collected after 0, 60, 180 min and 20 h.

To block the reaction SDS loading buffer 1X (Bio-Rad) +0.7 M β-mercaptoethanol was added to each aliquot which was promptly heated at 90 °C for 3 minutes. Proteolysis reaction was monitored by SDS-PAGE.

### Limited proteolysis

WT and N184K G2l were dissolved at 1 mg/ml in PBS, pH 7.4. Trypsin (PROMEGA) was added to the sample at a concentration of 2 ng/μl at room temperature. Proteolysis samples (10, 20, 30, 60, 90, 120 minutes) were analysed by SDS-homogeneous 15% PAGE (GE Healthcare).

### Fibrillogenesis

WT and N184K G2l were diluted 1 mg/ml in either 50 mM Sodium Acetate, pH 5.5 or 50 mM Sodium citrate, pH 2.5. 10 μM Thioflavin T (ThT) was added to the sample and aliquots of 100 μl each were loaded in Costar 96-well black-wall plates then covered with sealing films. The plates were incubated at 37 °C and subjected to 900 rpm double-orbital shaking using a BMG LABTECH FLUOstar Omega plate reader. Thioflavin T fluorescence emission at 480 nm was monitored by bottom reading using 445 nm as excitation wavelength and fluorescence was monitored in three or more replicates for each well until it reached a plateau.

### Crystallization, structure solution and analysis

Crystallization trials were performed at 20 °C using an Oryx-8 crystallization robot (Douglas Instruments) and several commercial screens in a sitting-drop set up: 0.2 or 0.3 μl of 8 mg/ml N184K G2s in 20 mM HEPES, 300 mM NaCl, pH 7.4, supplemented with 5 mM CaCl_2_ were mixed with 0.2 or 0.1 μl of the reservoir solution, respectively. Best diffracting crystals were obtained in either 200 mM calcium acetate, 100 mM MES pH 6.0, 20% PEG 8000 (orthorhombic form) or 0.2 M lithium sulfate, 0.1 M TrisHCl pH 8.5, 40% PEG 400 (trigonal form). The former was cryoprotected in a reservoir solution supplemented with 20% glycerol before being flash-cooled in liquid nitrogen. X-ray diffraction data were collected at beamline ID29 (ESFR, Grenoble) at 100 K. Data were processed with iMOSFLM^21^ and scaled with SCALA^22^.

N184K G2s crystal structure was solved by molecular replacement with the program PHASER^23^, using G2 wt structure (pdb ID 1KCQ) as searching model. Structures were refined with both refmac5^24^ and Phenix refine^25^ and manual model building performed with Coot^26^. Pymol was used to both analyse the structures and prepare the figures^27^.

### Molecular dynamics simulation

The molecular dynamics simulations were performed using the program NAMD2^28^. Briefly, we started from the orthorhombic structure of N184K and added 6 missing amino acids at the C-term (261–266) in random conformation resulting in a model composed of 118 amino acids (from Gly148 to Ala266) and a Ca^2+^ ion. In order to produce the model for wt simulation we modified the same pdb introducing the mutation K184N.

Using the program psfgen (part of the namd2 package^28^) H-atoms were added and partial charges assigned to every atom in the two models. Then for each model a box was built around the protein (67.1 × 69.4 × 66.6 Å^3^) and filled with 8,892 and 8,896 water molecules in N184K or wt, respectively. The charge equilibration of the two systems was performed adding NaCl 0.2 M (37/36 Cl^−^ and 34/34 Na^+^ ions, in N184K/wt using the vmd package; http://www.ks.uiuc.edu/Research/vmd/). Initially, harmonic constraints to fix protein atom positions during energy minimization of the solvent were applied for 0.5 ns, before equilibrating the systems at 310 K for additional 0.5 ns. The simulation was then started, and lasted 40 ns, with 2 fs time steps. During the simulation periodic boundary conditions in NPT ensemble, with Langevin temperature control (T = 300 K) and Langevin piston Nose-Hoover method^29^, were used to maintain constant temperature and pressure, respectively. Van der Waals interactions were handled with a cutoff of 12 Å, and switched off using a smoothing function beyond 10 Å. The electrostatic interactions were treated with the Particle Mesh Ewald method^30^, using a grid of 100 points along each dimension of the simulation box.

## Additional Information

**Accession codes:** Atomic coordinates and structure factors for the N184K variant of gelsolin G2 domain were deposited in the Protein Data Bank, with accession codes 5FAF and 5FAE.

**How to cite this article**: Bonì, F. *et al*. Molecular basis of a novel renal amyloidosis due to N184K gelsolin variant. *Sci. Rep.* **6**, 33463; doi: 10.1038/srep33463 (2016).

## Supplementary Material

Supplementary Information

## Figures and Tables

**Table 1:** Summary of the thermal stability experiments.

	G2l wt	G2l D187N	G2l N184K	G2s N184K
	Tm (°C)	Tm (°C)	Tm (°C)	Tm (°C)
+Ca	60.6	46.5	45.6	47.0
−Ca	41.5	46.4	33.4	33.8
ΔTm	19	0	12	13

Tm values (±0.3 °C) are reported for the G2l construct of the wt protein and mutants D187N and N184K and the short construct of mutant N184K. Measurements were performed in the presence of either 1 mM calcium chloride (+Ca) or 1 mM EDTA (−Ca).

**Table 2:** Data collection and refinement statistics.

PDB ID	Orthorhombic	Trigonal
	5FAF	5FAE
Data collection		
Beamline	ID29 (ESRF)	ID29 (ESRF)
Wavelength (Å)	1.0	1.0
Space group	P22_1_2_1_	P3_1_21
Cell dimension a, b, c (Å); α, β, γ (°)	26.4, 50.1, 80.2; 90.0, 90.0, 90.0	46.9, 46.9, 84.6; 90.0, 90.0, 120.0
Unique reflections	48782	12405
Resolution range (Å)	42.50–1.05 (1.11–1.05)^+^	40.63–1.70 (1.73–1.70)^+^
I/σ(I)	8.3 (2.4)^+^	7.5 (2.1)^+^
Rmerge (%)	5.0 (76.2)^+^	10.4 (79.3)^+^
Completeness (%)	96.8 (91.4)^+^	99.9 (100)^+^
Multiplicity	3.5 (3.0)^+^	6.9 (7.2)^+^
Refinement		
Resolution range (Å)	26.40–1.05 (1.07–1.05)^§^	26.45–1.70 (1.77-1.70)^§^
R_work_/R_free_^*^(%)	13.0/16.8 (41.1–42.5)^§^	18.2/22.5 (27.0–31.0)^§^
RMSD		
Bonds (Å)	0.023	0.018
Angles (°)	2.172	1.538
Ramachandran plot		
In preferred regions(%)	96	96
In allowed regions(%)	4	4
Outliers(%)	0	0
B-factors (Å^2^)^#^	24	34

^+^ and ^§^ Values in parentheses refer to the highest resolution shells.

*Rwork = Σhkl||Fo|−|Fc||/Σhkl|Fo| for all data, except 5–10%, which were used for Rfree calculation.

^#^Average temperature factors over the whole structure.
