# Supplementary material for: Molecular basis of a novel renal amyloidosis due to N184K gelsolin variant
Source: Sci Rep. 2016 Sep 16;6:33463. doi: 10.1038/srep33463 (PMC5025852; doi:10.1038/srep33463)
Supplement: Supplementary Information [file srep33463-s1.pdf]

Supplementary

Molecular basis of a novel renal amyloidosis due to N184K gelsolin variant

Francesco Bonì[1,2], Mario Milani[1,2*], Riccardo Porcari[3], Alberto Barbiroli[4], Stefano Ricagno[2*] and Matteo de Rosa[1,2*]

[1]CNR Istituto di Biofisica, c/o Dipartimento di Bioscienze, Università degli Studi di Milano, 20133 Milano, Italy
[2]Dipartimento di Bioscienze, Università degli Studi di Milano, 20133 Milano, Italy
[3]Wolfson Drug Discovery Unit, Centre for Amyloidosis and Acute Phase Proteins, University College London, London NW3 2PF, UK
[4]Dipartimento di Scienze per gli Alimenti, la Nutrizione e l'Ambiente, Università degli Studi di Milano, 20133 Milano, Italy
* corresponding authors (mario.milani@unimi.it, stefano.ricagno@unimi.it, teo.derosa@gmail.com)

```
          133                 151                                184 187
           |                   |                                  |   |
G2l   YFKSGLKYKKGGVASGFKHVVPNEVVVQRLFQVKGRRVVRATEVPVSWESFNNGDCFILDLGNNIHQ
G2s   ------------------HVVPNEVVVQRLFQVKGRRVVRATEVPVSWESFNNGDCFILDLGNNIHQ

G2l   WCGSNSNRYERLKATQVSKGIRDNERSGRARVHVSEEGTEPEAMLQVLGPKPALPAGTEDTAKEDAA
G2s   WCGSNSNRYERLKATQVSKGIRDNERSGRARVHVSEEGTEPEAMLQVLGPKPALPAGTEDTAKEDAA
                                                                        |
                                                                       266
```

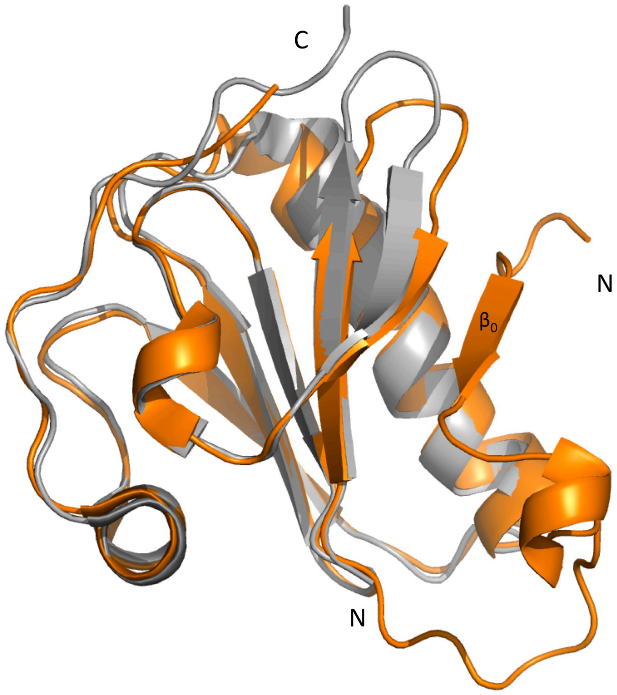

Figure S1: Definition of gelsolin domain 2. Sequence alignment of the two constructs used for this study (named N184K G2l and G2s respectively) and superimposition of the crystal structure of the stand alone gelsolin domain 2 wt as obtained by Kazmirski et al 2002 (grey, pdb ID 1KCQ) and extrapolation from the crystal structure of full-length inactive (Ca^{2+}-free) gelsolin (orange, pdb ID 3FFN) of the stretch comprising residues 133-266.

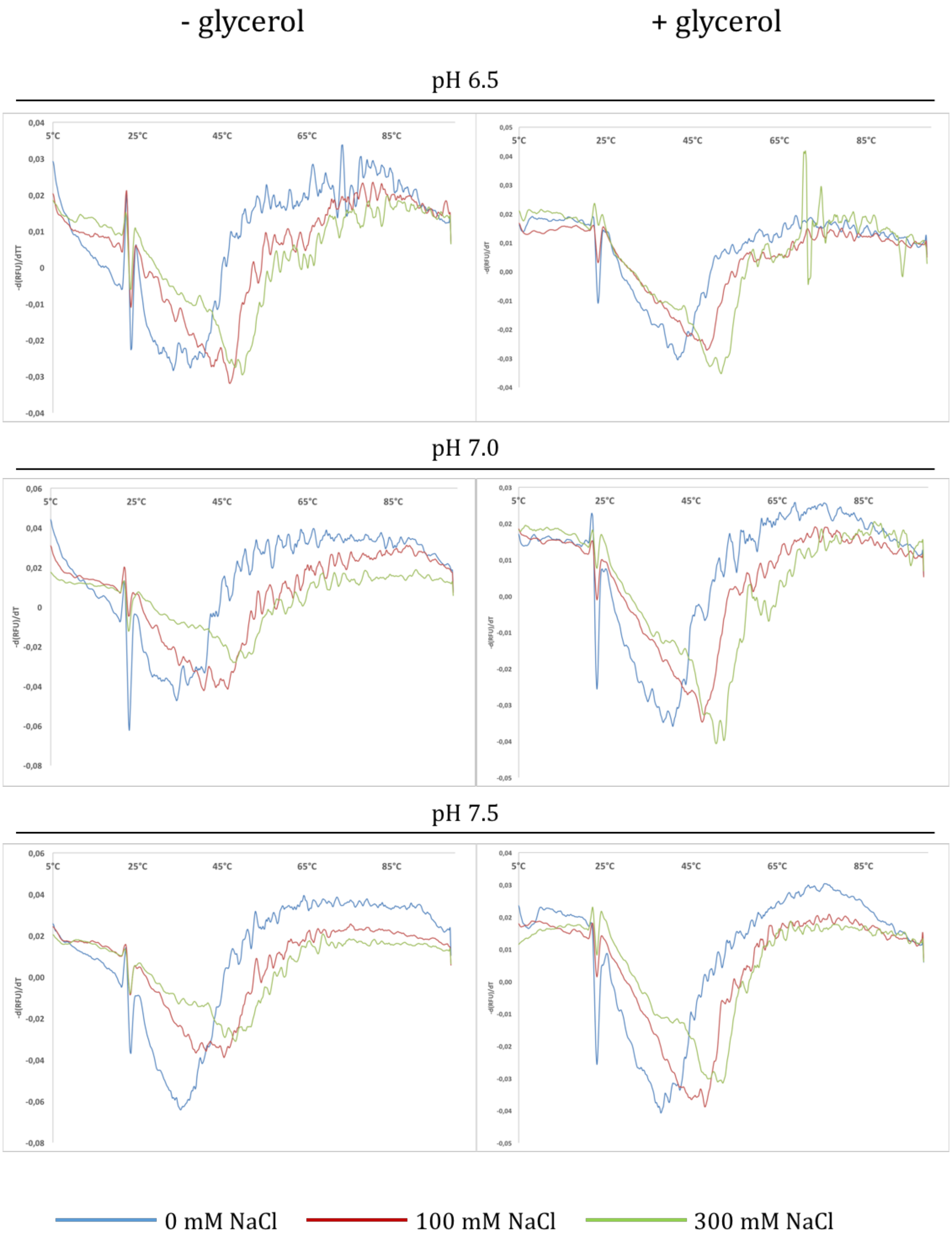

Figure S2 Thermodynamic stability of N184K G2l assessed by thermo-fluorimetry assay. In the graph the first derivative of SYPRO orange fluorescence is reported as function of the temperature in different pH and ionic strength conditions. In addition, the effect of 10% glycerol was tested.

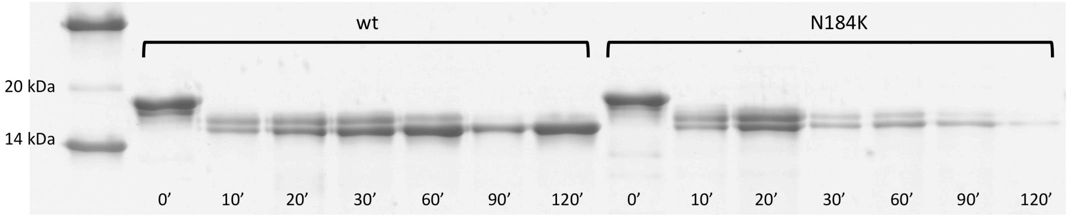

Figure S3: Kinetics of the proteolysis of wt and N184K G2l by trypsin. Both variants show a similar proteolytic pattern for the intact domain (first 20' window), suggesting that the mutant does not possess an overall enhanced flexibility.

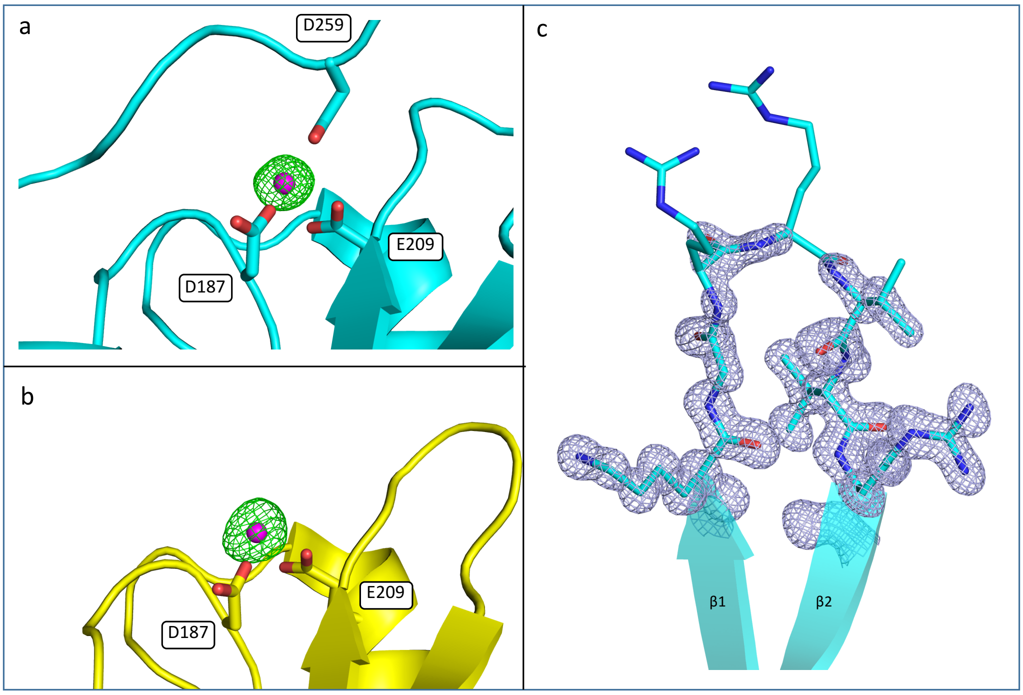

Figure S4 Details of the N184K structures **a,b)** Fo-Fc omit map (3 σ) calculated excluding the calcium ion of the orthorhombic (cyan) and trigonal (yellow) crystal form. **c)** electron density map (2Fo-Fc, 1.5 σ) of the β1-β2 loop in the orthorhombic structure.

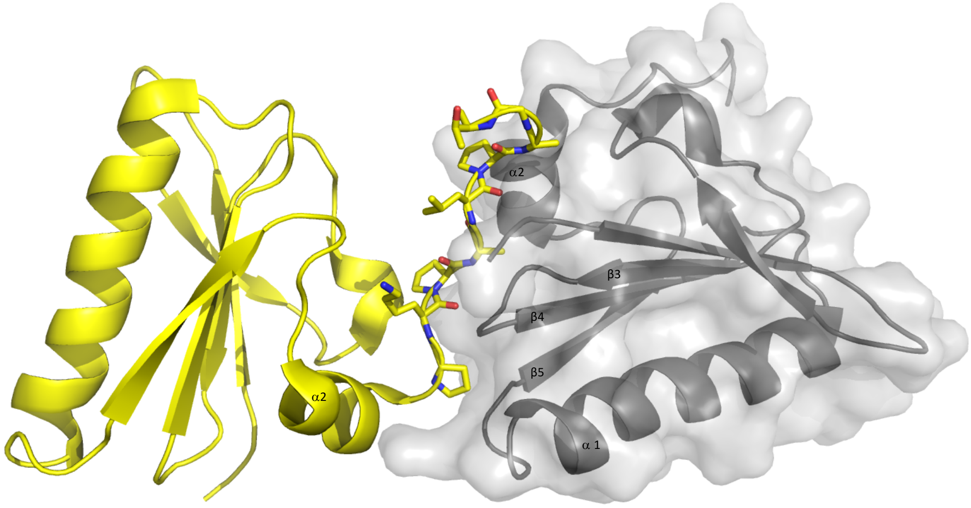

Figure S5 Interactions of the C-*terminus* in the trigonal structure with a symmetrical molecule. In this crystal form, the terminal tail is found in an "open" conformation, partly stabilized by the crystal packing.

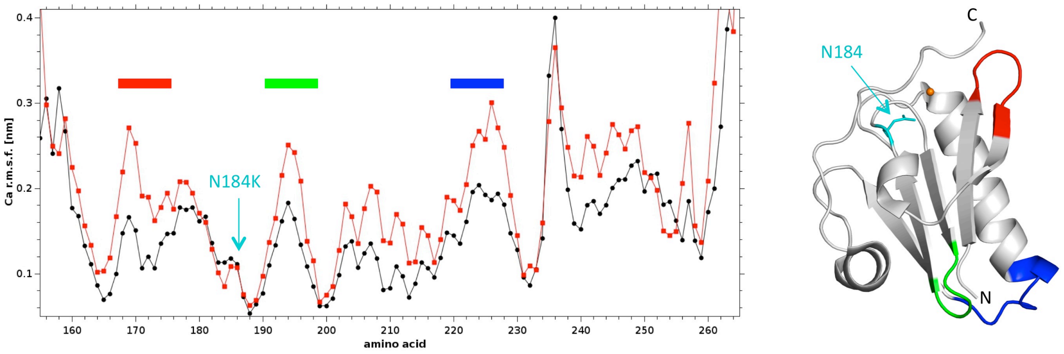

Figure S6 Comparison of the dynamic behavior of G2 wt (black trace) and N184K (red trace). Stretches of the N184K variant showing a significant higher flexibility are marked and mapped on the crystallographic structure of the protein.
